# Incidentally Detected Colloid Cyst in a Pediatric Patient With Facio-Audio-Symphalangism Syndrome: A Case Report

**DOI:** 10.7759/cureus.105999

**Published:** 2026-03-27

**Authors:** Emad M Babateen, Hydar AlQassab, Ayman Bahatheq

**Affiliations:** 1 Neurosurgery, Security Forces Hospital, Riyadh, SAU; 2 Medicine, King Saud University, Riyadh, SAU

**Keywords:** colloid cyst, facio-audio-symphalangism syndrome, pediatrics, sudden death, third ventricle

## Abstract

Colloid cysts are rare, benign intracranial lesions typically arising from the anterosuperior third ventricle. Although commonly diagnosed in adults, their occurrence in pediatric patients is uncommon and may follow a more aggressive clinical course, occasionally resulting in acute deterioration or sudden death. The association between colloid cysts and genetic syndromes remains poorly understood. We report the case of a nine-year-old girl with facio-audio-symphalangism syndrome in whom a third ventricular colloid cyst was incidentally discovered during routine surveillance imaging. The patient was neurologically stable and asymptomatic with no clinical features of raised intracranial pressure. Neuroimaging revealed a well-circumscribed lesion at the foramen of Monro with imaging characteristics consistent with a colloid cyst measuring 1.2 × 1.0 × 0.8 cm and mild ventricular enlargement. Genetic testing demonstrated a heterozygous mutation in the *NOG* gene. After multidisciplinary discussion and extensive counseling with the family regarding the risks of observation versus surgical intervention, elective resection was performed via a right interhemispheric transcallosal approach. Gross total resection was achieved without complications. At the three-year follow-up, the patient remained clinically stable with no radiological evidence of recurrence. Pediatric colloid cysts are rare, and their incidental discovery in asymptomatic children is particularly uncommon. The association with facio-audio-symphalangism syndrome in this case is unusual and could be associated with a possible genetic contribution. Given the uncertain natural history in children, individualized management and shared decision-making remain essential.

## Introduction

Colloid cysts are benign lesions that are typically located in the anterosuperior portion of the third ventricle [1]. These cysts are thought to arise from endodermal embryonic remnants of the brain, normally situated in the anterior roof of the third ventricle [1]. As gelatinous mucoid material accumulates and cyst size increases, symptoms may emerge, typically due to pressure exerted on the foramen of Monro during the third to fifth decades of life, which leads to obstructive hydrocephalus and high intracranial pressure symptoms [1]. The estimated annual occurrence of symptomatic colloid cysts is approximately 3.2 cases per million individuals [2]. Despite 1,167 documented cases reported in the literature between 1858 and 1995, colloid cyst detection during childhood is infrequent, accounting for merely 1%-2% of all documented instances [2]. Notably, there are few reports of pediatric colloid cyst cases and series, underscoring the importance of characterizing this condition in this demographic. Such reporting is critical for advancing understanding of pediatric clinical presentations, management strategies, and outcomes, thereby facilitating improvements in diagnostic accuracy and treatment approaches tailored to children. Here, we report a case of a nine-year-old female patient with facio-audio-symphalangism syndrome who presented with a colloid cyst of the third ventricle, which was incidentally found on imaging. 

## Case presentation

A nine-year-old girl with a known diagnosis of facio-audio-symphalangism syndrome was evaluated during routine follow-up. She was neurologically stable and asymptomatic, with no headache, nausea, vomiting, visual changes, or other signs suggestive of increased intracranial pressure. The patient had speech delay and behavioral dysregulation consistent with the neurodevelopmental manifestations of her underlying syndrome. Cranial imaging for surveillance demonstrated an incidental, well-circumscribed hyperintense lesion in the anterior third ventricle at the foramen of Monro, radiologically compatible with a colloid cyst.

Radiological findings

**Figure 1:**
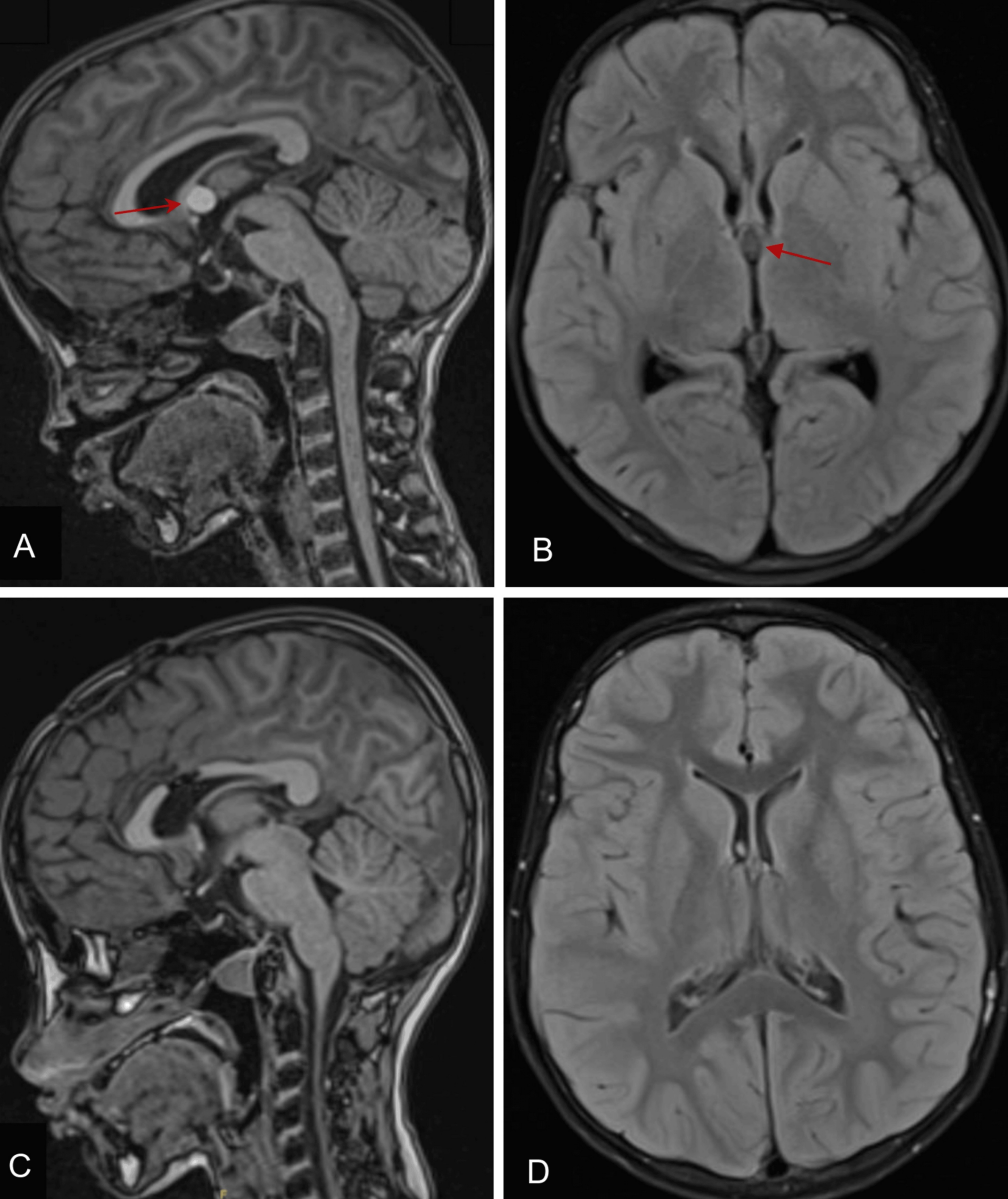
Preoperative MRI demonstrating a third ventricular colloid cyst, and postoperative MRI confirming complete resection. (A) Preoperative sagittal T1-weighted MRI showing a cyst that is hyperintense to the CSF within the third ventricle (red arrow). (B) Axial preoperative view. (C) Postoperative sagittal T1-weighted MRI showing the successful resection of the colloid cyst and restoration of the CSF flow. (D) Axial postoperative view. CSF, cerebrospinal fluid; MRI, magnetic resonance imaging.

Computed tomography revealed a small, roughly rounded, well-defined hyperdense lesion measuring 9 × 10 × 11 mm in diameter at the anterior aspect of the third ventricle near the foramen of Monro. No dilatation of the ventricles or significant mass effect was observed. Magnetic resonance imaging ​​showed a well-defined cystic lesion within the midline of the foramen of Monro, measuring 1.2 × 1.0 × 0.8 cm, with associated mild dilatation of the lateral ventricles. The cyst exhibited high signal intensity on T1-weighted imaging and low signal intensity on T2-weighted imaging, consistent with a colloid cyst. No periventricular edema was observed, and normal gray-white matter differentiation of both cerebral and cerebellar hemispheres was noted (Figure 1).

Genetic findings

Comprehensive genome sequencing identified a heterozygous likely pathogenic frameshift variant in the *NOG* gene (NM_005450.6: c.149_150del; p.Pro50ArgfsTer5), predicted to result in premature truncation of the protein. The variant was classified as likely pathogenic according to the American College of Medical Genetics (ACMG) criteria and is associated with autosomal dominant phenotypes.

Surgical approach

A right interhemispheric transcallosal approach was used. The right medial frontal lobe was retracted from the falx to expose the corpus callosum. A limited anterior callosotomy of approximately 1.5 cm in length was made in the body of the corpus callosum to access the right lateral ventricle. The choroid plexus was traced to the foramen of Monro, where the colloid cyst was identified, partially aspirated, and resected. Hemostasis was secured, and an external ventricular drain was placed under direct vision.

Postoperatively

The postoperative course was uneventful, with no neurosurgical complications. The patient remained at her preoperative neurologic baseline. Follow-up postoperative neuroimaging demonstrated complete resection of the cyst with expected postoperative changes (Figure 1).

## Discussion

Colloid cyst is a non-cancerous brain cyst located within the upper anterior part of the third ventricle, which has the potential to cause severe complications by obstructing the cerebrospinal fluid flow, leading to severe complications such as increased intracranial pressure, hydrocephalus, drop attacks, and, in rare cases, sudden death [3].

The occurrence of colloid cysts in pediatric patients is very rare, with few cases reported worldwide. Most colloid cyst presentations are asymptomatic and incidentally diagnosed. Based on the findings of the McCrea study, it was estimated that 67% of the pediatric patients are asymptomatic at the time of diagnosis [3], and according to the largest series to date, including 134 pediatric cases of colloid cysts, patients become symptomatic after the age of 10 years, and headache is the most common presenting symptom [4,5]. 

Numerous studies have documented that colloid cysts in pediatric patients tend to exhibit a more aggressive clinical course than in adult patients, as they may lead to sudden death [3,4]. However, few reports have documented sudden death occurrences associated with colloid cysts [5]. The cause of these sudden deaths was identified through biopsies or postmortem examination [6-8]. In a review of 21 reported cases of sudden death, seven of them were under the age of 18 years [9]. 

Early diagnosis and prompt intervention, especially in pediatric patients, yield substantial benefits, including reduced mortality rate, improved overall survival rate, and minimized risk of recurrence [3].

While our case was found to have a genetic comorbidity, the limited number of documented cases and the variation of clinical presentations complicate the identification of the genetic basis underlying the condition. Some published reports have highlighted familial patterns in colloid cysts, suggesting an inherited predisposition [10]. These familial occurrences suggest that genetic factors play an important role in the pathophysiology of colloid cysts. Those previous results may indicate the presence of an autosomal dominant inheritance pattern in some cases [10].

Surgical intervention is the primary treatment modality for colloid cysts, with several indications, including the presence of symptoms and the size and growth of the cyst [3]. In adults, the Colloid Cyst Risk Score has been developed and independently validated to stratify the likelihood of symptomatic presentation and hydrocephalus using five clinical and radiological criteria (age <65 years, headache, cyst diameter ≥7 mm, FLAIR hyperintensity, and location within a risk zone), with scores ≥4 identifying a high-risk subgroup that may benefit from surgical intervention or closer surveillance [11]. In a study conducted by Roth et al., approximately 77% of patients underwent surgery, and 60% of those underwent endoscopic resection [4]. Among the children who were initially observed after diagnosis, roughly 50% required urgent surgical intervention due to developing new symptoms [4]. In a review of four pediatric cases who underwent transcallosal-transforaminal excision and were monitored for a mean follow-up duration of 23.2 months, the patients showed no instances of tumor recurrence on subsequent imaging examinations throughout the entire follow-up period [12]. Our patient was followed for three years post-surgical resection with a repeated MRI brain, which showed no sign of tumor recurrence.

Small incidental colloid cysts have frequently been documented. One study stated that patients with small cysts who exhibit no symptoms or signs of hydrocephalus and who are managed conservatively remain asymptomatic during extended follow-up periods [13]. Pollock and Huston retrospectively analyzed cases of third ventricular colloid cyst and found no symptomatic progression at 2 and 5 years and an 8% progression rate at 10 years [14]. No sudden deaths were reported during the follow-up period [13]. 

Maqsood et al. reviewed pediatric colloid cysts and noted that acute deterioration or death was typically preceded by days to months of headache and symptoms of raised intracranial pressure, often associated with hydrocephalus. Although sudden deterioration was uncommon, its risk was unpredictable. Based on documented cyst growth and recurrence, the authors advocated complete surgical excision in children [15].

## Conclusions

The results of our study suggest that genetics play a potential role in pediatric colloid cysts, highlighting the necessity of more focused investigations into the genetic basis of colloid cysts in pediatric patients. Future studies should systematically analyze genetic factors associated with the development of colloid cysts, especially in pediatric patients. These studies could provide valuable insights into the etiology and the risk of occurrence, as well as enhance surgical approaches.

## References

[1] Maeder PP, Holtås SL, Basibüyük LN, Salford LG, Tapper UA, Brun A (1990). Colloid cysts of the third ventricle: correlation of MR and CT findings with histology and chemical analysis. AJNR Am J Neuroradiol.

[2] Hernesniemi J, Leivo S (1996). Management outcome in third ventricular colloid cysts in a defined population: a series of 40 patients treated mainly by transcallosal microsurgery. Surg Neurol.

[3] McCrea HJ, Lara-Reyna J, Perera I (2021). Colloid cysts of the third ventricle in children. J Neurosurg Pediatr.

[4] Roth J, Perekopaiko Y, Kozyrev DA, Constantini S (2022). Pediatric colloid cysts: a multinational, multicenter study. An IFNE-ISPN-ESPN collaboration. J Neurosurg Pediatr.

[5] Vazhayil V, Sadashiva N, Nayak N, Prabhuraj AR, Shukla D, Somanna S (2018). Surgical management of colloid cysts in children: experience at a tertiary care center. Childs Nerv Syst.

[6] Goyal N, Sharma BS, Mahapatra AK (2014). Third ventricular colloid cysts in children - a series of eight cases and review of the literature. Turk Neurosurg.

[7] Alzahrani A, Albouijan A, Alshamsi G, Almanjumi A, Hamdi M, Alteraiqi B, Alshaikhi M (2023). Sudden unexpected death caused by a colloid cyst in the third ventricle: case report. Egypt J Forensic Sci.

[8] Turillazzi E, Bello S, Neri M, Riezzo I, Fineschi V (2012). Colloid cyst of the third ventricle, hypothalamus, and heart: a dangerous link for sudden death. Diagn Pathol.

[9] Alnaghmoosh N, Alkhani A (2006). Colloid cysts in children, a clinical and radiological study. Childs Nerv Syst.

[10] Calderón C, Fernandez-de Thomas RJ, De Jesus O (2020). Familial colloid cysts of the third ventricle: case report and literature review. Asian J Neurosurg.

[11] Alford EN, Rotman LE, Shank CD, Agee BS, Markert JM (2020). Independent validation of the colloid cyst risk score to predict symptoms and hydrocephalus in patients with colloid cysts of the third ventricle. World Neurosurg.

[12] Kapu R, Symss NP, Pande A, Vasudevan MC, Ramamurthi R (2012). Management of pediatric colloid cysts of anterior third ventricle: a review of five cases. J Pediatr Neurosci.

[13] Desai KI, Nadkarni TD, Muzumdar DP, Goel AH (2002). Surgical management of colloid cyst of the third ventricle--a study of 105 cases. Surg Neurol.

[14] Pollock BE, Huston J 3rd (1999). Natural history of asymptomatic colloid cysts of the third ventricle. J Neurosurg.

[15] Maqsood AA, Devi IB, Mohanty A, Chandramouli BA, Sastry KV (2006). Third ventricular colloid cysts in children. Pediatr Neurosurg.

